# Letter to the Editor: The 5WH framework: A practical tool for addressing symptoms

**DOI:** 10.1016/j.fhj.2026.100531

**Published:** 2026-04-02

**Authors:** Jason W. Boland, Catherine D'Souza, Elaine G. Boland

**Affiliations:** Wolfson Palliative Care Research Centre, Hull York Medical School, University of Hull, UK; Palliative Care Medical Director, South Canterbury District Health Board, New Zealand; Hull University Teaching Hospitals NHS Trust, Cottingham, East Yorkshire, UK

**Keywords:** Symptoms, Pain, Nausea, Vomiting, Assessment

## Abstract

• The 5WH framework enables a structured assessment for all clinical symptoms.
• It facilitates rapid and holistic symptom evaluation.
• 5WH reduces reflex prescribing by addressing specific underlying causes.
• It supports individualised, aetiology-driven clinical management plans.
• 5WH is a scalable tool to improve bedside decision-making and patient safety.

*Dear editor*,

We read with great interest the article by Theresa Barnes regarding the urgent need for a change in managing enduring symptoms.^1^ Barnes rightly highlights that all clinicians have, or need, awareness of symptoms and that there is increased distress to patients when there is no simple structural explanation for these symptoms. Inadequate assessment leads to poorly managed symptoms; this can occur for all symptoms, even those with, and especially those without, a simple structural explanation.

To assist this ‘call to action’ to be successful at the bedside, clinicians require a structured symptom assessment framework. While pain sometimes receives rigorous attention, other symptoms, such as nausea, vomiting or expectoration, are frequently managed without thorough assessment and with reflex prescriptions that might address the sensation, but overlook the aetiology.^2^ This not only risks pharmacological toxicity, but also fails to address the ‘holistic’ drivers that Barnes identifies as central to patient experience.^1^, ^3^

All symptoms need a structured assessment, to understand of the likely cause (and if an underlying cause is found, treating the underlying cause if it is appropriate), followed by non-pharmacological interventions and, when needed, targeted pharmacological treatment. These steps must be guided by a comprehensive individualised assessment. Although there are multiple frameworks for pain assessment, there is a lack of easily usable tools for assessing many other symptoms.

To operationalise the approach that Barnes advocates, we propose the adaptation of the 5WH (who, what, when, where, why and how) question model,^4^, ^5^ as a standardised tool for rapid, multidimensional symptom assessment in clinical practice. There is a focus on emesis for this example, but this could apply to any symptom (Box 1).

Box 1 **The 5WH (who, what, when, where, why and how) question model, with a focus on emesis for this example.**

• **Who:** Is understanding the patient, their family/social setting, disease(s), prognosis.
• **What:** Is assessment and differentiation of what is being described (nausea, vomiting, expectoration, regurgitation), and nature of the symptom (what is being vomited; type of vomit, blood etc).
• **When:** Identifies timing, triggers (including medication) and associated emotional components (eg fear/anxiety).
• **Where:** Contextualises the environment and relational triggers where the symptom occurs; within the place, people (family, friends, clinicians), environment where it is occurring (including if any of these are triggers/mediators for the symptom).
• **Why:** Investigates underlying pathophysiology through physical examination and targeted investigation.
• **How:** Assesses the functional impact on the patient/family and dictates a co-produced holistic management plan.

Depending on this brief assessment, more detailed evaluations might be required. Management plans must be reviewed for both effectiveness as well as potential harm, and plans adapted accordingly.

By adopting this practical framework, healthcare systems can move toward the ‘immediate action’ that Barnes calls for, ensuring that assessment is both thorough and aetiology-driven before pharmacological intervention is initiated. This framework offers a scalable, low-cost intervention to reduce the diagnostic inertia that currently characterises the management of enduring symptoms.

## Declaration of competing interest

The authors declare that they have no known competing financial interests or personal relationships that could have appeared to influence the work reported in this paper.
